# Not all who wander are lost: prospecting and settlement of male floaters in the spotless starling

**DOI:** 10.1093/beheco/araf028

**Published:** 2025-04-17

**Authors:** Iraida Redondo, Roger Fusté, Jaime Muriel, Eduardo Gómez-Llanos, Raquel Monclús, Lorenzo Pérez-Rodríguez, Diego Gil

**Affiliations:** Departamento de Ecología Evolutiva, Museo Nacional de Ciencias Naturales (MNCN), CSIC, José Gutiérrez Abascal 2, E-28006 Madrid, Spain; Departamento de Ecología Evolutiva, Museo Nacional de Ciencias Naturales (MNCN), CSIC, José Gutiérrez Abascal 2, E-28006 Madrid, Spain; Departmento de Zoología, Universidad de Córdoba, Edificio Charles Darwin, Campus de Rabanales, 14071 Córdoba, Spain; Department of Biology, University of Turku, Vesilinnantie 5, 20500 Turku, Finland; Departamento de Ecología Evolutiva, Museo Nacional de Ciencias Naturales (MNCN), CSIC, José Gutiérrez Abascal 2, E-28006 Madrid, Spain; Laboratoire d’Ethologie Expérimentale et Comparée UR 4443, Université Sorbonne Paris Nord, 93430, Villetaneuse, France; Instituto de Investigación en Recursos Cinegéticos (IREC), CSIC-UCLM-JCCM, Ronda de Toledo 12, 13005, Ciudad Real, Spain; Departamento de Ecología Evolutiva, Museo Nacional de Ciencias Naturales (MNCN), CSIC, José Gutiérrez Abascal 2, E-28006 Madrid, Spain

**Keywords:** floaters, prospecting, public information, spotless starling, territory

## Abstract

Floaters are non-breeding individuals that lack a territory or a breeding site. In many species, they can be seen visiting the territories of conspecifics before obtaining their own breeding site. Prospecting behavior is hypothesized to benefit floaters through information acquisition, enhanced site familiarity and dominance over other floaters. Here, we used detections of PIT-tagged male floaters in a population of spotless starlings (*Sturnus unicolor*). We investigated how floater activity varied across breeding stages and how their visits influenced subsequent nest site selection. We also tested whether distance, reproductive success, and phenotype and fate of the former owner influenced final settlement. We found that floater activity increased during the nestling-rearing period as nestling age increased. Floaters were more likely to breed near the area where they had been detected the previous year, suggesting that prospecting allows males to secure a foothold in their future settlement area. Although prospecting was higher in nests with a higher number of nestlings, neither breeding success, phenotype, nor provisioning rate of the last owner were related to nest choice, suggesting that public information is not used by males to decide where to settle. However, we found that floaters were more likely to breed in nest boxes where the previous owner had disappeared from the colony, suggesting that visits by male floaters in this species allow them to detect new vacancies. Our results suggest that prospecting might serve several non-mutually exclusive functions. Further studies in non-saturated colonies could shed light on the functional aspects of prospecting.

## Introduction

Choosing a place to breed constitutes a decisive moment in the life of every individual. During this time, dispersing individuals may restrict their movements to assess the relative quality of different sites before deciding where to settle (Delgado et al. 2009). If all breeding sites are occupied and further dispersal becomes too risky, dispersing individuals may benefit from exploring sites that could become available in the future. These individuals that do not acquire a breeding site and remain in the population are known as floaters (Penteriani et al. 2011). Floaters are non-territorial but sexually mature individuals that coexist with breeders during the breeding season and are a ubiquitous component of avian populations (Brown 1969; Moreno 2016). Despite not defending a territory or a breeding site, floaters can often be detected within a relatively confined zone intruding into the territories of other conspecifics (Smith 1978; Tobler and Smith 2004).

It has been suggested that intrusions made by floating individuals may serve several non-mutually exclusive purposes. Firstly, floaters could obtain fitness benefits through alternative reproductive tactics (ie extrapair copulations, intraspecific brood parasitism; Kempenaers et al. 2001; Saitou 2001; Tucker et al. 2016). Secondly, floaters might accumulate local dominance to more easily acquire a territory in the event of the owner’s disappearance. This hypothesis has been coined by different authors as the “queue” hypothesis (Ens et al. 1995), the “site dominance” hypothesis (Sergio et al 2009), or the “foothold” hypothesis (Piper et al. 2015), and requires that floater prospecting areas match later settlement areas. Thirdly, birds could gather public information to either assess potential competitors and ease the posterior eviction of the former owner (ie assessment hypothesis; Arcese 1987) and/or gather information on the presence and reproductive performance of conspecifics (Valone and Templeton 2002), or the availability of vacancies (Smith, 1978; Zack and Stutchbury 1992). Public information provides reliable knowledge about the quality of an area since it integrates information from both environmental and social factors, as long as they are predictable over time (Boulinier and Danchin 1997). Visits intended for gathering public information are referred to as “prospecting” (Reed et al. 1999).

Among these three hypotheses, the public information hypothesis has received the most support. Indeed, it is well known that prospectors increase their activity during the nestling rearing period (Stutchbury and Robertson 1987; Schjørring et al. 1999; Tobler and Smith 2004; Veiga et al. 2012), when information on reproductive performance is expected to be more reliable and of higher value, and visit more frequently nests with larger broods and higher parental provisioning rates (Doligez et al. 2004; Parejo et al. 2008; Schuett et al. 2017; Brandl et al. 2019). Although it has been demonstrated that individuals of some bird species settle or disperse as a function of the average reproductive success of a given patch (Danchin et al. 1998; Doligez et al. 2002; Parejo et al. 2007; Boulinier et al. 2008), fewer studies look at how individual prospecting behavior relates to subsequent individual settlement. However, in some species, prospectors have been shown to settle close to previously visited successful sites (Pärt and Doligez, 2003; Ward 2005; Pärt et al. 2011). The second hypothesis, eg gaining a higher site-dominance by concentrating their activity within a limited area (Smith, 1978), is supported by work in several bird species, including waterbirds and passerines, and also in *Anolis* lizards (Smith 1987; Stamps 1987; Heg et al. 2000; Piper 2011; Piper et al. 2015).

The spotless starling (*Sturnus unicolor*) is a relatively long-lived hole-nesting passerine that breeds in loose colonies. Male spotless starlings typically start to breed as two- or three-year-old adults (Veiga et al. 2012; Redondo et al. 2022). Individuals that do not acquire a nest remain as floaters in the population and often visit active nest boxes owned by other breeding pairs (Parejo et al. 2008; Veiga et al. 2012), as does its sister species, the European starling, *Sturnus vulgaris* (Sandell and Diemer 1999; Tobler and Smith 2004). However, studies have not investigated how intrusions relate to subsequent nest site choice in these species. The main difficulty in this respect lies in the logistic demands involved in the long-term monitoring of the elusive floaters. However, radio-frequency identification (RFID) technology allows us to record the prospecting activity of marked floaters, providing an excellent opportunity to investigate how these intrusions relate to subsequent nest site choice.

In this study, we present long-term data (10 breeding seasons) on the intrusion behavior and posterior nest site choice of male floaters in a population of spotless starlings. Females were not considered because data available on prospecting for this sex was scant. In this study, our objective is two-fold. Firstly, to describe the temporal patterns of prospecting in this species by monitoring nest intrusions by tagged individuals and examine the cues that may induce birds to prospect in particular boxes. Secondly, we test the main assumptions of the different hypotheses that have been proposed to explain prospecting (see above). Our predictions were as follows:

(1) The alternative reproductive hypothesis would require that most floaters are detected slightly before egg laying, since extra-pair copulations can only be successful while the female is fertile (Birkhead and Møller, 1998). Our test of the temporal dynamics of floating allows us to test the general relevance of this hypothesis.
(2) The foothold hypothesis would predict that floaters occupy boxes where they had prospected in previous years.
(3) The public information hypothesis makes a series of specific predictions, depending on whether the information gathered is reproductive success, competitor fighting ability or vacancy availability. In the first case, we would expect to find important levels of temporal and spatial autocorrelation in reproductive success, and male floaters to visit nests with larger brood sizes (Pärt and Doligez 2003). In addition, we expect breeding site choice to be influenced by previous parental provisioning rates and reproductive success. In the second case, we expect floaters to preferentially settle in boxes where the previous owner was younger and showed reduced ornamentation, and body condition. And in the third case, we expect settlement to be preferential in nests where the previous owner fails to survive over the non-breeding period.

## Material and methods Study species & study area

The spotless starling is a sedentary semi-colonial passerine distributed throughout the western Mediterranean (Craig et al. 2020). Spotless starlings are strict secondary cavity nesters that readily occupy nest boxes, and their territory consists of a small area surrounding the nest. They are gregarious and breed in proximity to each other during the breeding season. The species is facultative polygynous, although most males are monogamous in this population (Celis et al. 2021). Males are bigger than females and have a blueish patch in the base of the beak as well as longer and modified ornamental throat feathers (Hiraldo and Herrera 1974). Throat feathers become longer with age (Hiraldo and Herrera, 1974) and their length is related to body condition and other proxies of individual quality (Aparicio et al. 2001; Gil and Culver 2011; Ruiz-Rodríguez et al. 2015).

In our study population, the breeding season spans from March to July. During this period, females can lay up to 2 clutches. If the first clutch is lost, females can lay a replacement clutch. Modal clutch size is 4 eggs (D’Arpa et al. 2022). Incubation is mainly carried out by females and lasts around 12 d. Both parents participate in the feeding of the chicks, but females generally show higher and more consistent levels of parental care (authors’ unpublished data; Jimeno et al. 2014). Fledglings leave the nest when they are about 23 d old (Jimeno et al. 2014).

Male and female starlings differ in their recruitment age, with males only exceptionally breeding as 1-y olds (Redondo et al. 2022). Due to the limited availability of cavities for nesting, competition for nesting holes is high. There are both male and female floaters, but the former are more numerous as shown by capture-recapture analyses (Gil et al. in prep). Floaters from both sexes are often detected visiting active nests of conspecifics during the breeding season, especially during the nestling rearing period (Veiga et al. 2012, 2013; Gómez-Llanos et al. 2024).

This study was conducted in a population of spotless starlings breeding in a fully- occupied set of 246 nest boxes distributed in an open mixed ash (*Fraxinus angustifolia*) and oak (*Quercus pyrenaica*) woodland of approximately 80 ha in central Spain (Soto del Real, Madrid, ca. 40° 45′ N, 3° 48′ W).

### Field methodology

About one month before the onset of egg-laying, we captured adult individuals sleeping in their nest boxes by blocking the entrance of the nest box before sunrise. We deployed spring traps during the rest of the morning (08:00 to 12:00) to capture visiting individuals. All individuals caught during these capture sessions were marked with a unique numbered metal ring and were implanted with a passive integrated transponder tag (hereafter, PIT-tag) that contains a unique alphanumeric code (ID-100 Trovan Unique Implantable tags, Trovan Ltd, UK). For every captured individual, we measured body mass with a digital scale (accuracy = 0.1 g, Ohaus, Model CS200, Pine Brook, New Jersey), tarsus and beak length with a digital caliper (accuracy = 0.01 mm, Mitutoyo Absolute, Japan) and wing length with an end-stop ruler (accuracy = 1 mm). We also plucked three ornamental throat feathers from each individual and measured their length in the lab with a digital caliper (accuracy = 0.01 mm, Mitutoyo Absolute, Japan). Given that throat feather length shows high repeatability (Redondo et al. 2022), we used the mean length of the three measurements as an estimate of ornament size.

By the beginning of April, we checked every nest box in the colony until we found the first laid egg. At that moment, capture sessions ended, and we started visiting each nest box every 1 to 2 d until clutch completion. Nest boxes were visited again 12 d later to record the hatching date. After hatching, we checked every nest box when chicks were 3, 6, 14, 18 and 25 d old to quantify nestling survival and record nest breeding success, calculated as the number of nestlings that fledge. Nestlings were marked with a metal ring and a PIT-tag when they were 14 d-old.

We deployed PIT-tag readers (LID-650 decoder, Trovan Ltd, UK) to record the visitors’ ID as well as the time and date of the visits. PIT-tag readers are deployed throughout the breeding season to record the activity of breeders and floaters during: (1) the prelaying period, (2) the laying-incubation period and (3) the nestling rearing period. PIT-tag readers are deployed for 1.5 to 2 d at each of these stages. Readers are placed sequentially in batches of nest boxes until we record the whole colony in both first, replacement and second clutches. During 2019 and 2020, we set up a network of 30 solar-powered RFID readers distributed across the study site programmed to work between 07:00 to 13:00 each morning (due to battery limitations we favored this period which corresponds to the peak of starling nest visits).

We identified the owners of each nest box using the data collected by readers. Briefly, we established specific criteria for each sex to determine nest box ownership. Female breeders are identified by using a hand-held PIT-tag reader at night (GR250, Trovan) during incubation and also with parental visits obtained during the chick-rearing period. In the case of males, the owner is established as the most frequent male visitor before breeding and during chick feeding (feeding a minimum of 20% of the number of female feeds).

Data collected by the readers also allowed us to count and identify floaters. We considered floaters those individuals that were detected by capture or PIT-tag readings in the population but not recorded breeding in any nest box throughout the whole breeding season. Due to the existence of some natural holes in our field area, a small percentage of our presumed floaters could be nesting in natural cavities. However, the number of natural holes in our study area is small and unlikely to have a strong effect in our classification (0.57 natural nests/Ha vs. 3.3 nest boxes/Ha I. Redondo unpublished data).

All applicable institutional and national guidelines for the care and use of animals were followed. Permission to capture and manipulate birds were authorized by the Consejería de Medio Ambiente (Comunidad de Madrid, Spain) under licence from the Spanish institutional authorities (Consejería de Medio Ambiente and Centro de Migración de Aves de SEO/BirdLife). The permit to insert PIT-tags was granted from the Dirección General de Agricultura de la Comunidad de Madrid (PROEX 201/18).

### Prospecting patterns

We gathered data collected from the solar-powered RFID readers during the 2019 and 2020 nesting seasons. Overcast skies caused some disruptions in data collection, so we considered only days in which a minimum of 120 min were recorded in a given nest box (range: 120 to 361 min, mean = 299.1, SD = 17.9). We selected those breeding events (N = 82) in which both parents were PIT-tagged, and where RFID data was available for a minimum of eight days during the nestling period. We also included data from the pre-laying (14 d before laying), egg laying, incubation, and fledging periods (19 to 22 d old). From the broods that did not fledge offspring (12/82: 14.6%), we only included data of the periods when nestlings were known to be alive.

The yearly breeding status of each bird was scored according to the following criteria: (1) birds that bred in the first clutch wave (first broods) were considered breeders for the remainder of the year; (2) birds that were detected breeding in subsequent clutch waves (intermediate and second broods) were considered transitioning floaters before that breeding event, and breeders after it. Birds that were never detected breeding in a given year were considered floaters *sensu stricto*. In the final analysis, all floaters (transitioning and *sensu stricto* floaters) were pooled into a single category.

### Prospecting and subsequent nest site choice

For the analysis of the relationship between the prospecting area and subsequent nest site choice, we selected data from 8 cohorts of males (2011 to 2019) born in our population. We selected individuals detected as 1-y-old and/or 2-y-old floaters and subsequently as 2-y-old and 3-y-old breeders. These individuals had been marked with a PIT-tag since birth or since they were 1 y old, and thus, we were able to confirm that they had not bred in any of the colony’s nest boxes. Those individuals that were not trapped as adults were sexed by means of a molecular technique using their stored blood samples (Griffiths et al. 1998).

From PIT-tag readings during the breeding season, we identified the nest boxes that were prospected by our focal birds when they were 1 and 2 y old (n = 124 and n = 15, respectively). We chose for the analysis those males that were detected visiting a minimum of 5 different nest boxes during their year as floaters (mean number of visited nest boxes = 15; range = 5 to 46). With the spatial coordinates of the visited nest boxes, we calculated the minimum convex polygon 95% (MCP) and its associated centroid point for each male starling using the packages *adehabitatHR* (Calenge 2006) and *geosphere* (Hijmans 2021). We chose the median area from all the calculated MCPs as the less biased estimate of home range and drew a circle of the same area with its center on the centroid point of each individual. We included in the analysis all nest boxes contained in this circle, since we assumed that males would have ample time to visit them during a reproductive season. We compared the characteristics of this set of presumed visited boxes with those of the nest box where they eventually bred. We used these measurements of reproductive success: (1) annual number of young fledged, (2) young fledged in the second brood, ie the last breeding window of the previous season, and (3) whether the nest box was or not successful in its last breeding attempt (considering a nest box successful if at least one young fledged). We also included some characteristics of the former male owner, including: age, ornamentation (mean throat feather length), body condition, and provisioning rate during the second broods. Lastly, we also monitored the fate of the former owner of each nest box, classifying them as “detected again” or “not detected again” in the colony. We decided to consider the last owner of a nest box, since it is to be expected that floaters use the reproductive information from this last reproductive attempt rather than from previous ones.

### Statistical analysis

#### Prospecting patterns

Data were analyzed using R v. 4.0.3 (R Core Team 2020). We used generalized linear mixed models from the package *glmmTMB* (Brooks et al. 2017) to analyze factors influencing the number of floaters visiting a nest box. On the first model, we assessed differences in number of floaters in relation to breeding stage of the nests. Explanatory variables included: breeding stage (a factor with 7 levels: pre-laying, laying, incubation and nestling rearing period which was divided according to nestling age in 4 periods (1 to 7 d old, 8 to14 d old, 15 to 18 d old and 19 d old-fledging time), clutch wave (a factor with two levels: 1: first broods, 2: intermediate and second broods) and year. The duration of the recording bouts (in hours) was fitted in the model as an offset variable. We fitted the model with a Poisson error distribution and a log link function. On the second model, we investigated which variables influenced the number of floaters visiting a nest during the nestling stage. Explanatory variables were the following: age of nestlings and its quadratic term, the number of parental visits, the clutch wave, the number of chicks and the year. Nest box identity was included as a random effect. The duration of the recording bouts (in hours) was also fitted as an offset variable. For this model, we fitted a hurdle model with a zero-truncated Poisson distribution. Hurdle models are itemized in a conditional part that describes the expected rate of non-zero counts and a zero-inflated part that models the probability of a zero count.

#### Temporal and spatial autocorrelation

For reproductive success to become a reliable proxy for a site’s quality, reproductive success must be predictable over time (Boulinier and Danchin 1997). We checked for temporal autocorrelation of reproductive success within each nest box, considering the annual number of young fledged in each nest box as a proxy of reproductive success. We excluded from this analysis boxes subjected to experimental manipulations.

We also checked for spatial autocorrelation in reproductive success to ascertain the degree of local patchiness in the number of fledglings in the population using the Moran’s I index (Moran 1948; Cliff and Ord 1973). Moran’s I is a commonly used index in this type of analysis, with values ranging from -1 and to 1. Positive values indicate that nests that are close to each other tend to have similar reproductive success, whereas negative values would indicate the opposite, indicating, for instance, breeding inhibition between neighbors (Marrot et al. 2015). For the calculation of the Moran’s I, we selected data from years in which nest boxes were not subjected to manipulations and averaged the number of fledglings raised across the years.

#### Prospecting and subsequent nest site choice

We calculated the Euclidean distance between the centroid of the prospected area and the location of the nest site that was chosen in the following breeding season using the *geosphere* package (Hijmans 2021). To exclude the possibility that male floaters settle in areas close to the nests where they were born, we also calculated the natal dispersal distance (calculated as the Euclidean distance between the birth nest and the breeding nest) as a proxy for philopatry. We compared it with the distance between the prospected area and the breeding nest. We assessed if these distances differed from those generated by a random process using a permutation test. We carried out 10,000 permutations (N = 139) in which we calculated the distance between each centroid of the prospected area and a random nest box from the colony, and between each birth nest and a random nest box from the colony. For each permutation we calculated the mean of the distance and compared it with the mean of the observed dataset. P-values were calculated as the percentage of cases in which the mean of the observed data was smaller than the permuted means (percentile method). The same method was used to assess whether the proportion of birds breeding in nest boxes within the prospected area was significantly different from random. We also carried out 10,000 permutations (N = 139) in which a nest box was randomly sampled. We checked if a given nest box was included in the prospected area of each individual. Lastly, we summed up the number of nest boxes that were included in the prospected areas and compared it with the observed value using the percentile method.

To analyze the factors associated with the selection or not of a nest, we used generalized linear mixed models (GLMM) using the package *lme4* (Bates et al. 2015). We analyzed the probability that a nest was chosen for breeding in relation to several variables related to reproductive performance and information about the last owner using univariate GLMMs with a binomial distribution and a logit link function. We considered as predictors: (1) the annual number of young fledged in a nest box the year previous to breeding, (2) whether the nest was or not successful (1 to 0) during the second broods (coded as 1 when at least one young fledged), (3) the age, (4) ornamentation (5) and body condition of the last male owner, and (6) the mean provisioning rate of the last male owner during the second broods.

For the calculation of body condition, we first calculated an index of body size using a principal component analysis (PCA) on three morphological traits: beak, wing, and tarsus length. We took the first principal component (PC1), in which all loadings were positive (beak: 0.60, wing: 0.53 and tarsus: 0.60). The PC1 had an Eigenvalue of 1.32 and explained 44% of the variance. Once we obtained this body size index, we calculated body condition as the residuals of a linear mixed model (LMM) where body mass was the response variable and the body size index (PC1), time, and date of capture were the predictors. In this model, the year and the identity of the individual were included as random effects.

Provisioning rates were calculated as the number of times per hour a male and a female entered their nest box during the nestling rearing period after applying an 8 s filter to avoid multiple detections. To account for the influence of brood size and age of the nestlings in the provisioning rate variable, we built a linear mixed model using the package *lme4* (Bates et al. 2015). Brood size but not nestling age influenced the mean provisioning rate, so we removed the variable age from the model. Year was included as a random effect in the model. The residuals from this model were used as a proxy of the mean provisioning rate corrected by brood size.

We also built a last model in which we explored if nest site choice was associated with the return to the breeding grounds of its last owner. We chose to use univariate models because the high percentage of NAs in the data led to a small sample size if all variables were considered in a single model. In all these models, the identity of floaters and the year were included as random effects. Due to the presence of missing data about the identity and phenotypic characteristics of the last male owners, the sample size of each model varied. For all models, we verified that the assumptions of normality and homocedasticity of residuals were met using the *DHARMa* package (Hartig 2021).

## Results

### Prospecting patterns

The number of male floaters detected in a nest increased as breeding progressed, with more floaters detected during the nestling rearing period compared to the pre-laying stage, laying, and incubation (Table 1). Nest boxes during the nestling rearing period had many more male visitors than during the prelaying period. For instance, in the case of first broods –those with the highest floater activity, the difference amounted to 23% more visitors when nestlings were 1 to 7 d old, 46% for 8 to 14 d old nestlings, 53% for 15 to 18 d old nestlings and 45% for > 18 d old nestlings. This corresponds to an increase from virtually no visiting floaters in early breeding stages to a mean of 3 floaters per hour during the nestling care period (Table 1). The same pattern was observed in broods from the second wave (Table 1).

**Table 1.** Results of the generalized linear mixed model investigating the number of different male floaters visiting breeding active nest boxes in relation to breeding stage. The model was fitted with a Poisson error distribution and log link function.

Random effects	σ^2^	SD			
Nest ID	0.111	0.333			
Fixed effects	β	SE	Wald χ^2^	P value	CI 95%
Intercept	−2.465	0.199	-		
Stage [Laying]	−0.117	0.284	**274.476**	**<0.001**	−0.672 to 0.439
Stage [Incubation]	0.066	0.280			−0.482 to 0.614
Stage [1 to 7 d old]	1.016	0.200			**0.624** to **1.408**
Stage [8 to 14 d old]	1.695	0.193			**1.317** to **2.074**
Stage [15 to 18 d old]	1.838	0.194			**1.457** to **2.220**
Stage [> 18 d old]	1.683	0.207			**1.276** to **2.089**
Wave [Second wave]	−0.235	0.096	**5.999**	**0.014**	−**0.424** to **(-0.047)**
Year [2020]	0.008	0.094	0.007	0.934	−0.177 to 2.089

P-values were calculated via ANOVA type III. The reference level for factors Stage, Wave and Year are “Pre-laying,” ‘First wave and “2019” respectively. N = 971. Statistically significant results are presented in bold.

**Fig. 1.**
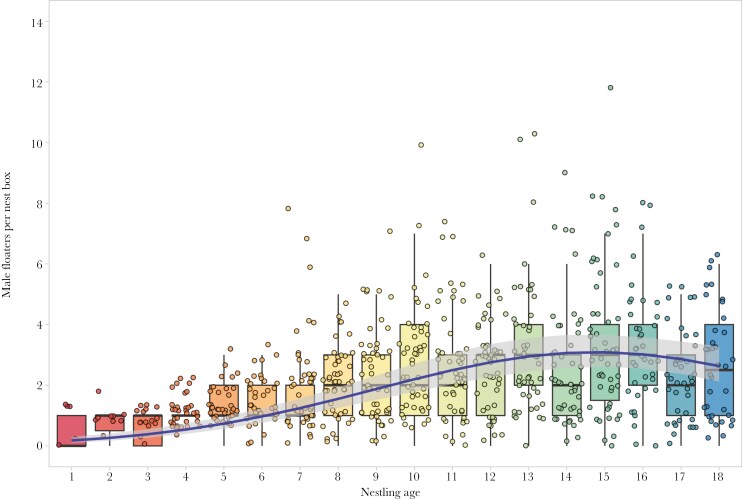
Boxplot of the number of different male floaters visiting nest boxes during the nestling rearing period. The blue solid line represents the model prediction. The grey area around the blue time corresponds to the 95% confidence intervals.

Nestling age and the number of nestlings were positively associated with the number of male floaters visiting occupied nest boxes (Table 2). For the zero-inflated part of the hurdle model, our results showed that the probability of detecting male floaters increased during the late part of the chick-rearing period (Table 2, Fig. 1).

**Table 2.** Results of the hurdle model investigating the number of different male floaters visiting breeding active nest boxes during the nestling stage. The model was fitted using a truncated Poisson error distribution and log link function.

	Zero inflated part				Conditional part			
Random effects	σ^2^	SD			σ^2^	SD		
Nest ID	0.787	0.887			0.101	0.318		
Fixed effects	β	SE	Wald χ^2^	*P* value	β	SE	Wald χ^2^	*P* value
Intercept	−1.600	0.886	-		−1.875	0.253	-	
Nestling age	−8.626	4.069	**17.007**	**<0.01**	15.821	1.373	**137.050**	**<0.01**
Nestling age^2^	10.045	3.614			−8.032	1.277		
Parent visits	0.001	0.004	0.017	0.625	0.001	0.001	2.632	0.105
Number of nestlings	−0.273	0.173	2.492	0.121	0.132	0.046	**8.061**	**<0.01**
Wave [Second wave]	0.560	0.394	2.025	0.122	−0.061	0.120	0.253	0.615
Year [2020]	−0.273	0.173	0.002	0.962	0.068	0.108	0.394	0.530

P-values were calculated via ANOVA type III. The reference level for factors Wave and Year are “First broods” and “2019” respectively. N = 719. Statistically significant results are presented in bold.

#### Temporal and spatial autocorrelation

We found that the reproductive success within each nest box, measured as the total number of young fledged from a nest box in a year, was moderately positively autocorrelated between consecutive years, but not at larger time lags (time lag = 1; autocorrelation coefficient = 0.25; Fig. S2)

A positive and significant but very weak spatial autocorrelation for reproductive success was detected only in the most proximal lag (estimate = 0.10, SD = 4.35, *P* < 0.01; Fig. S3).

#### Prospecting area

We followed the settlement of a total of 139 male floaters in our 80-ha study site over 8 y-cohorts. Of these birds, 124 (89%) were floaters only during their first year as adults, and the remaining 15 males (11%) were floaters during their first and second years as adults. The median area of the MCP 95% was 4.46 ha ± 6.34 SD, and we used that median to project the prospecting circles from the centroid points.

**Fig. 2.**
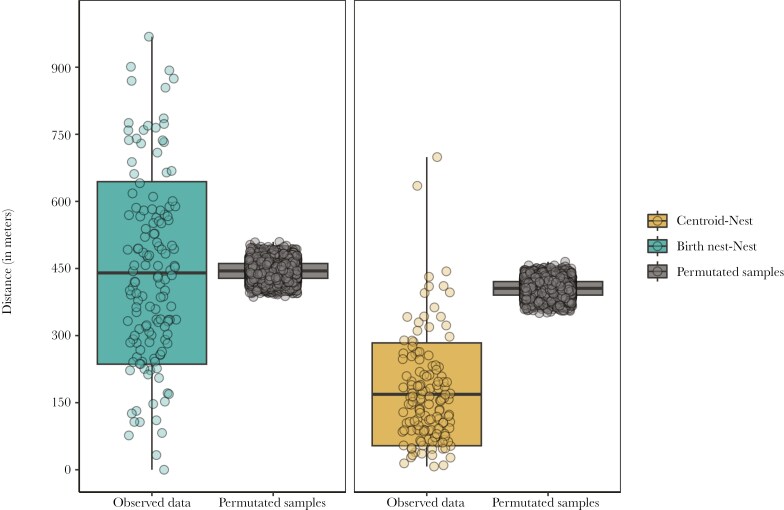
Boxplot of distances between the birth nest and subsequent breeding nest (left panel, blue boxplot) and between the centroid of the prospecting area and subsequent breeding nest (right panel, gold boxplot). Grey boxplots represent the distribution of the permuted distances.

The mean distance between the prospecting centroid and the breeding nest was 168.68 m (range: 7 to 699 m). This distance was approximately four times smaller than the mean random distance between the centroids and all nest boxes of the colony (406 m, range: 355 to 465 m) (Fig. 2), indicating that first-year prospecting area strongly determined settlement patterns (permutation test, *P*= < 0.001). Natal dispersal distance (birth nest to adult nest) was greater than the distance between the adult nest and the prospecting centroid (440 m; paired t-test: −14.123, d.f = 138, *P* < 0.001, Fig. 2), and not different from random distances within the area (permutation test, *P* = 0.709, Fig. 2)

Despite the fact that RFID monitoring was haphazard and limited in time, 13% (18/139) of floaters were detected breeding in a nest box where they had been detected in the previous year. When considering nest boxes embedded in the prospecting area, this number raised up to 39% (54/139), which greatly differed from random generated values (permutation test *P* < 0.001; Fig. S1).

#### Factors influencing subsequent nest-site occupancy

We did not find evidence of any association between the nest occupied by a male floater and the variables related to reproductive success of the last male owner (Table 3). We neither detected an association between the nest occupation and the reproductive success when this was simplified into a 2-level categorical variable (at least one young fledged vs. zero young fledged; Table 3).

**Table 3.** Results from the generalized linear models assessing the association of choosing a nest box as a function of previous year reproductive success and the return and phenotypic traits of the last male owner.

Univariate models	β	SE	Odds Ratio (95% CI)	*P* value
Annual young fledged (N_males_ = 139; N_obs_ = 2494)	−0.06	0.04	0.94 (0.86 to 1.02)	0.142
Second brood young fledged (N_males_ = 139; N_obs_ = 2494)	−0.11	0.07	0.90 (0.78 to 1.03)	0.127
Second brood not succeeded (N_males_ = 139; N_obs_ = 2494)	0.16	0.10	1.17 (0.98 to 1.40)	0.077
Age (N_males_ = 139; N_obs_ = 210)	0.13	0.13	1.14 (0.89 to 1.15)	0.317
Ornamentation (N_males_ = 139; N_obs_ = 456)	−0.04	0.05	0.96 (0.88 to 1.05)	0.351
Body condition (N_males_ = 139; N_obs_ = 455)	0.05	0.27	1.05 (0.61 to 1.79)	0.861
Feeding rate (N_males_ = 139; N_obs_ = 129)	0.13	0.08	1.14 (0.97 to 1.33)	0.110
Owner not returned (N_males_ = 139; N_obs_ = 661)	0.70	0.14	**2.01 (1.53 to 2.67)**	**<0.01**

Each row of the table corresponds to a univariate model following a binomial distribution of errors and a logit link function. N_males_ informs about the number of focal males that were included in the model. N_obs_ reports the total number of rows forming each dataset. Note that the sample size in the models about the phenotypic characteristics of the former owner varies due to the presence of missing data. Statistically significant results are presented in bold.

In relation to the phenotypic characteristics of the last male owner of the nest box (body condition, ornamentation or provisioning rate), we did not find evidence that any of them were associated with the occupation or not of a nest box (Table 3). However, we found that the likelihood of occupying a nest was positively associated with the absence of its previous owner (Table 3). In other words, settlers were more likely to breed in a nest if the previous owner had not survived.

## Discussion

In some species, non-territorial birds intrude into conspecifics territories or nests before acquiring one themselves. Despite being documented in many species, our knowledge about this behavior is generally poor. In this study, we investigated the prospecting behavior of floaters and how it related to subsequent nest choice in the spotless starling and assessed the relevance of different hypotheses to explain the intrusions.

The number of male floaters prospecting increased as breeding progressed, with a higher number of male floaters visiting nest boxes during the nestling rearing period, a pattern previously found in other species (Schjørring et al. 1999; Doligez et al. 2004). The fact that virtually no male floaters were found during the pre-laying and the laying stages speaks against the possibility that male floaters were using prospections to pursue extra-pair copulations (Birkhead and Møller, 1998). Although we cannot dismiss the possibility that some male floaters do gain some reproductive benefits at this time, our data shows that most prospecting occurs when this alternative strategy is no longer viable.

We found that during the breeding season, the home range prospected by male spotless starlings in their early floating years was small. Male floaters inspected multiple potential sites in these areas (Parejo et al. 2008; Veiga et al. 2013), and finally settled in nest boxes that were closer to their prospecting areas than to their natal nests. In addition, 40% of floaters settled in nest boxes that were included within their prospecting area. These findings suggest that prospective movements in the years before settling play a relevant role in breeding site acquisition. Our results resemble those found for other species, in which floaters acquired cavities/territories that they had previously intruded (Common and Barrow’s goldeneye (*Bucephala clangula, Bucephala islandica*): Eadie and Gauthier 1985; Stutchbury 1991); Purple martin (*Progne subis*): ) or territories that were close to their prospected areas (Song sparrow (*Zonotrichia capensis*): Smith 1978; Eurasian oystercatcher (*Haematopus ostralegus*): Bruinzeel and van de Pol 2004; Red-winged blackbird (*Agelaius phoenicus*): Yasukawa 1979; Northern wheatear (*Oenanthe oenanthe*): Pärt et al 2011). These settlement patterns are those expected under the foothold hypothesis, which predicts floaters to settle around the prospecting area (Smith, 1978; Piper et al. 2015).

However, although these findings strongly suggest that this behavior is related to breeding settlement, the specific aim of prospecting remains unknown and may serve several non-mutually exclusive functions in relation to obtaining public information.

Visits of floaters to active nests of other conspecifics have been suggested to be a way of gathering public information. Most studies so far focus on indexes of the reproductive success of conspecifics, by which prospectors may assess the quality of a site (Doligez et al. 2002), as long as this reproductive success is predictable in time. Our data from the continuous nest-box monitoring suggests that indeed floaters were more attracted to nest boxes with a higher number of nestlings. Although it is tempting to provide a functional explanation to this pattern (Parejo et al. 2008), assuming that floaters prefer to visit highly successful nests, it is not possible to discard the more parsimonious explanation that nests with more and larger nestlings are more likely to be detected because of the higher number of parental visits and higher levels of begging noise (begging in absence is particularly prevalent in those nests: Bulmer et al. 2008; Jimeno et al. 2014). There was only moderate temporal and spatial autocorrelation in reproductive success in our population, suggesting that male floaters may only gain a small advantage by targeting the most successful sites to breed in following years. Indeed, our analysis did not show any association between reproductive success of prospected nests and subsequent nest choice by male floaters. This speaks against the possibility that prospecting may be used by floaters to select breeding sites because of their inherent breeding success.

We also tested the possibility that floaters were more likely to settle in nest boxes with low male resource holding potential (low body condition or poor provisioning), which would be expected if prospecting allowed males to assess the capacity of breeders to defend their resource, but the contribution of these factors was not significant either. This was unexpected since a recent experiment in this population (Gómez-Llanos et al. 2024) showed that male floaters preferentially prospected nests in which male owners’ resource holding potential was reduced via wing-clipping. We predicted that prospecting would allow floaters to assess the condition and performance of owners and increase the chances of taking over a nest, maybe through direct aggressions or practicing a sort of “war of attrition” until the former owner decides to give up (Stutchbury 1991; Piper et al. 2000). However, our findings showed that owner’s resource holding potential was not a relevant factor for settlement patterns. How can we reconcile these two lines of conflicting evidence?

A mismatch between prospecting and settlement may arise if resource holding potential in a given year is not a predictor of future survival or if there are many contenders for a vacancy, thus lowering the chances of a prospector obtaining a box. In addition, although aggressive interactions are common in this species (pers. obs.), they entail costs for both breeders and floaters, sometimes even resulting in the death of one of the contenders. Given that most of the floaters considered in this study were young and inexperienced individuals with a low resource holding potential, it is unlikely that this is the main mechanism by which these floaters acquired a nest box (Shutler and Weatherhead 1991). Indeed, no differences were found in the number of takeovers between wing-clipped and non-wing-clipped males in the study conducted by Gómez-Llanos et al. (2024).

It is noteworthy that the single variable that predicted floater settlement was the disappearance of the previous owner, suggesting that vacancies may play a more relevant role in the acquisition of a breeding site. Vacancies are expected to be relatively common from year-to-year in species with low adult survival rates, and thus, they represent a good opportunity for floaters to obtain a nest box (Zack and Stutchbury 1992). In our population, male breeders have a relatively low mean survival rate that ranges between 0.54 to 0.60 (Redondo et al. in prep). Breeding male starlings are highly philopatric (authors’ unpublished data) and thus, breeding males that are no longer detected in the colony are most likely dead. This pattern gives weight to the idea that the information males seek while prospecting is nest-site availability.

It is important to consider that most studies that have found a relevant role of public information in prospecting behavior have dealt with populations where a good percentage of nest sites were not occupied. In contrast, in our spotless starling population, occupancy was complete. Given the intense competition for an essential and limiting resource (ie the cavity) and the absence of non-occupied nest boxes, it is likely that male floaters in our population are left with little room for choosing. In fact, when we added extra boxes in the middle of the breeding season, these were taken in a matter of days (authors’ unpublished data). This implies that the nest box is a limiting resource in our population that constraints the possibility of choice between different sites.

As proposed by other authors (Schuett et al. 2017), settlement decisions likely follow a hierarchical process in which dispersing individuals first identify successful patches to which they are attracted (by conspecific presence, number of young; Ward 2005; Calabuig et al. 2008) and then conduct small scale movements to select high-quality sites within a patch (Pärt and Doligez 2003). It is likely that the high annual productivity and the saturated scenario of our population primarily attract floaters to the woodland as a sign of good habitat quality. However, at smaller scale (ie the nest box), given the high uncertainty about the causes of nest failure, social information may be less valuable. Indeed, the spatial autocorrelation of reproductive success was weak, indicating that reproductive success is not spatially clustered in our colony. Moreover, all nest boxes are equal in size and are protected from predators with a metal cone. Thus, differences in quality between nest boxes must be small, perhaps related to heat exposure (Salaberria et al. 2014). These factors may suggest that reproductive success at the nest box scale may depend on stochastic processes or the quality of owners that do not confer relevant information at this scale.

We would argue that the most plausible scenario to account for our results is that by prospecting, floaters are able to detect the appearance of new vacancies more quickly, resulting in a greater probability of ensuring a site if it becomes suddenly available (eg death or abandonment of the former owner; Smith, 1978; Pärt et al. 2011). In addition, prospecting areas may allow floaters to accumulate competitive advantage for acquiring a nest (“foothold hypothesis,” Piper 2011). Through footholds, floaters could establish dominance relationships over other floaters that are also waiting for an opportunity to get a breeding site (Smith 1978; Zack and Stutchbury 1992; Bruinzeel and van de Pol 2004; Sergio et al. 2009). Thus, our results reinforce the idea that prospecting may be a way of securing future settling breeding site. However, whether the filling of the vacancies is opportunistic (eg the first floater that encounters a vacant site gets it) or it is the result of competitive contests cannot be ascertained with our data so far, and this remains an interesting question for future work.

In conclusion, we found that male floaters settled near the area where they had been detected prospecting the year before, suggesting a relevant role of prospecting in breeding site acquisition. Although floaters preferentially visited nests during the nestling rearing period and nests with a greater number of nestlings, reproductive success was unrelated to subsequent nest site choice. However, floaters settled in nests whose past owner was not seen again in the colony, suggesting an important role of prospecting in detecting vacancies towards successful breeding site acquisition. The intensive prospecting behavior of nest-sites by starlings gives us a unique window into the dynamics of floaters. Whether these patterns can be generalized to species in which territory quality is the key breeding resource is an open question, given the enormous logistic limitations of observing the discreet floaters while prospecting foreign territories.

## Supplementary Material

araf028_suppl_Supplementary_File

## Data Availability

Analyses reported in this article can be reproduced using the data provided by Redondo et al. (2025).
