# Supplementary material for: Not all who wander are lost: prospecting and settlement of male floaters in the spotless starling
Source: Behav Ecol. 2025 Apr 17;36(3):araf028. doi: 10.1093/beheco/araf028 (PMC12059212; doi:10.1093/beheco/araf028)
Supplement: araf028_suppl_Supplementary_File [file araf028_suppl_supplementary_file.docx]

**Online Supplement to**

**Not all who wander are lost: prospecting and settlement of male floaters in the spotless starling**

**Contents:**

**Figure S1. Histogram of the result of the permutation test to evaluate whether the number of floaters that bred in a nest box embedded in their prospecting area differed from random.**

**Figure S2. Temporal autocorrelation plot of the number of fledglings**

**Figure S3. Spatial autocorrelation plot of the mean number of fledglings**

**Figure S1.** Histogram showing the number of starlings breeding in a nest box within their prospecting area when nest box was randomly assigned to each individual using permutation sampling. Blue lines represent the 1.25% and 98.75% quantiles. The red line represents the observed number of starlings that bred in a nest box that was included in their prospecting area (n = 54). The number of permutated samples was 10000.


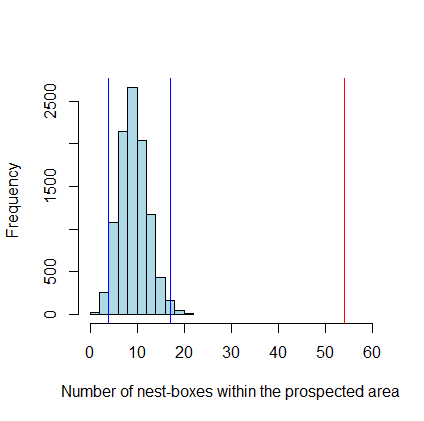


**Figure S2.** Autocorrelation plot showing the temporal autocorrelation in the number of fledglings within each nest box of the colony for a maximum lag of time of 5 years. Values of the temporal correlation correspond to the black dots. Purple dashed lines represent the 95% confidence intervals.

**
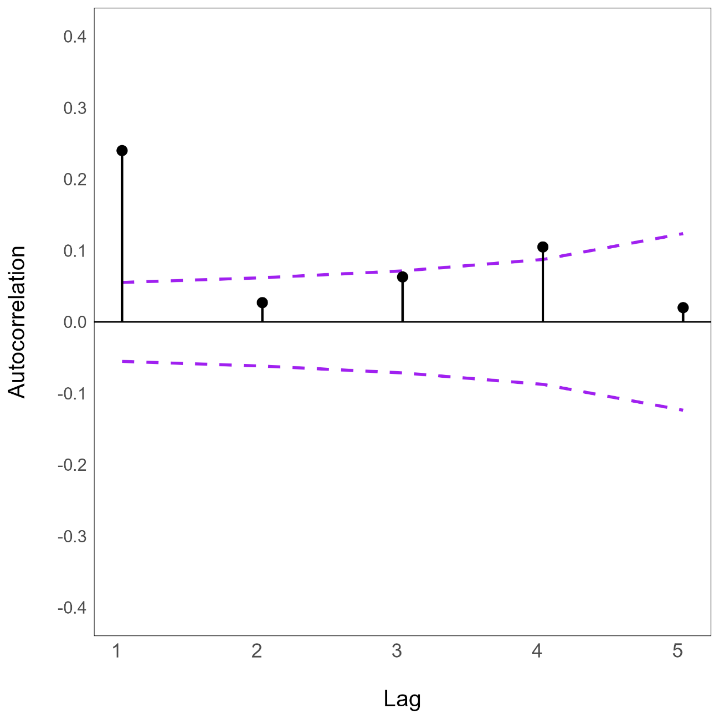
**

**Figure S3.** Moran’s I correlogram for mean number of fledglings at our starling colony. Moran’s I was calculated for 8 distance bands, from 125 m to 1081 m, with a lag distance of 125 m. Lag 1 showed a significant positive spatial autocorrelation.

**
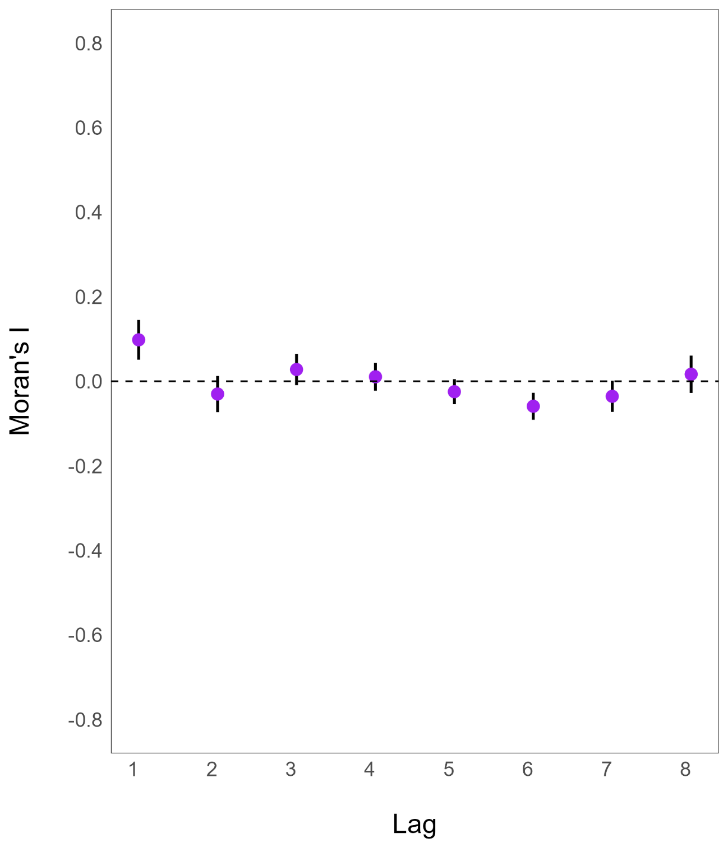
**
